# RIT2, a neuron-specific small guanosine triphosphatase, is expressed in retinal neuronal cells and its promoter is modulated by the POU4 transcription factors

**Published:** 2013-06-17

**Authors:** Ling Zhang, Karl Wahlin, Yuanyuan Li, Tomohiro Masuda, Zhiyong Yang, Donald J. Zack, Noriko Esumi

**Affiliations:** 1 The Wilmer Eye Institute, Johns Hopkins University School of Medicine, Baltimore, MD; 2 Departments of Neuroscience, Molecular Biology and Genetics, and McKusick-Nathans Institute of Genetic Medicine, Johns Hopkins University School of Medicine, Baltimore, MD; 3 Department of Ophthalmology, Shanghai First People's Hospital, Shanghai Jiao Tong University, Shanghai, China; 4 Institut de la Vision, Université Pierre et Marie Curie, 75012 Paris, France; 5 Montefiore Medical Center, 200 Corporate Boulevard, Yonkers, NY 10701

## Abstract

**Purpose:**

Ras-like without CAAX 2 (RIT2), a member of the Ras superfamily of small guanosine triphosphatases, is involved in regulating neuronal function. RIT2 is a unique member of the Ras family in that RIT2 is preferentially expressed in various neurons, including retinal neurons. The mechanisms that regulate RIT2 expression in neurons were studied.

**Methods:**

Reverse transcription-quantitative PCR (RT-qPCR), immunohistochemistry, western blotting, bioinformatic prediction, electrophoretic mobility shift assay (EMSA), and cell transfection methods were used.

**Results:**

With immunohistochemistry of the mouse retina, RIT2 protein was detected in the ganglion cell layer (GCL), inner plexiform layer, inner nuclear layer, and outer plexiform layer, with the strongest staining in the GCL and the inner plexiform layer. RT-qPCR combined with laser capture microdissection detected *Rit2* messenger RNA in the GCL and the inner nuclear layer. Western blot analysis showed a large increase in the RIT2 protein in the retina during maturation from newborn to adult. Transient transfection identified the 1.3 kb upstream region of human *RIT2* as capable of driving expression in neuronal cell lines. Based on the known expression pattern and biological activity, we hypothesized that POU4 family factors might modulate *RIT2* expression in retinal ganglion cells (RGCs). Bioinformatic analyses predicted six POU4 factor-binding sites within the 1.3 kb human *RIT2* promoter region. EMSA analyses showed binding of POU4 proteins to three of the six predicted sites. Cotransfection with expression vectors demonstrated that POU4 proteins can indeed modulate the human *RIT2* promoter, and that ISL1, a LIM homeodomain factor, can further modulate the activity of the POU4 factors.

**Conclusions:**

These studies confirm the expression of RIT2 in retinal neuronal cells, including RGCs, begin to reveal the mechanisms responsible for neuronal expression of RIT2, and suggest a role for the POU4 family factors in modulating RIT2 expression in RGCs.

## Introduction

The Ras superfamily of small guanosine triphosphate–binding proteins (small GTPases) comprises a large family of structurally related molecules that are involved in signal transduction and the regulation of a wide variety of cellular processes [1,2]. These small GTPases act as molecular switches for intracellular signaling cascades by alternating between an inactive guanosine diphosphate (GDP)–bound form and an active GTP-bound form [1,3,4]. At least five distinct families of the Ras superfamily have been defined: Ras, Rho, Rab, Arf, and Ran [1,2,4]. Within the Ras family, a distinct subclass is defined by mammalian Ras-like without CAAX1 (RIT1; formerly RIT) and RIT2 (formerly RIN) and *Drosophila* Ric [5-8]. RIT1 and RIT2 share many features of Ras, but they also demonstrate unusual structural characteristics, such as a distinct G2 domain and the absence of a CAAX motif for isoprenylation [5].

RIT1, like most of the Ras family members, is expressed ubiquitously; RIT2, however, is preferentially expressed in subsets of neurons, including retinal ganglion cells (RGCs) and selected neurons in the brain [5]. Accumulating reports suggest RIT2 has an important role in neuronal differentiation and function, and perhaps in neurological disease [9]. RIT2 couples stimulation by nerve growth factor to the p38 mitogen-activated protein kinase and v-raf murine sarcoma viral oncogene homolog B1 (BRAF) signaling pathways that are required for neuronal differentiation of PC6 pheochromocytoma cells [9]; RIT2 promotes neurite outgrowth through activation of Rac/Cdc42 and association with calmodulin [10]. RIT2 seems to be involved in downstream signaling of plexin B3, which stimulates neurite outgrowth of primary murine cerebellar neurons [11]. Pituitary adenylate cyclase-activating polypeptide 38, a potent neuropeptide, influences neuronal differentiation, and this effect is mediated, at least in part, by the Gα-Src-RIT2-HSP27 signal transduction pathway [12]. In addition, recent studies indicate a potential connection of RIT2 to human disease. Analyses of genome-wide copy number variation found that *RIT2* deletions were significantly overrepresented in schizophrenia cases [13]. In two patients with expressive speech delay, the smallest region commonly deleted at chromosome 18q12.3 contained *RIT2* as one of the likely candidate genes [14]. Furthermore, *Rit2* is highly and preferentially expressed in dopaminergic neurons in the substantia nigra [15], and meta-analysis of genome-wide association studies identified *RIT2* as a novel Parkinson disease susceptibility locus [16]. RIT2 directly interacts with the dopamine transporter and is required for its internalization and functional downregulation, which controls extracellular dopamine concentrations and half-life [17].

Based on the increasing evidence implicating RIT2 in neuronal differentiation and function, we became interested in the mechanisms regulating RIT2 neuron-specific expression in the retina, particularly in RGCs. Using a yeast two-hybrid screen, Calissano et al. found that RIT2 binds to the N-terminus of the POU4F1 transcription factor and modulated POU4F1-mediated activation of the Egr1 promoter [18]. These findings are of particular interest because the class IV POU domain (POU4, also known as BRN3) family of transcription factors is involved in the development, axonal growth, and pathfinding of RGCs and other sensory neurons [19-25]. In the eye, POU4F1 (also known as BRN3A), POU4F2 (BRN3B), and POU4F3 (BRN3C) are expressed in distinct but overlapping subsets of RGCs [19,21,26-29]. Although mouse knockouts indicate that POU4F1, POU4F2, and POU4F3 are essential for the development and survival of neurons in the trigeminal ganglia, RGCs, and auditory and vestibular hair cells, respectively [30-33], knock-in experiments indicate that these POU4 factors are largely functionally equivalent in terms of RGC development [34,35].

Here we report studies exploring the mechanisms regulating neuronal expression of RIT2. We identified a 5′-upstream region of human *RIT2* that demonstrates promoter activity preferentially in neuronal cells. Based on the studies cited above, we hypothesized that *RIT2* expression in RGCs might be modulated by POU4 proteins. Using a combination of bioinformatic analyses and biochemical promoter studies, we present data supporting this hypothesis.

## Methods

### Plasmid construction

To obtain human *RIT2* genomic clones, we screened a human genomic P1 library with *RIT2* cDNA probes following standard procedures [36]. Briefly, a P1 library that consisted of eight Southern membranes containing a high density array of DNAs (Genome Systems, St Louis, MO) was hybridized with ^32^P-labeled probes at 65 °C overnight, washed several times, and exposed to X-ray films. The films with positive spots were laid on the original membrane to identify the location of positive signals, and then positive clones were purchased (Genome Systems). *RIT2* promoter-luciferase reporter vectors were constructed with two human *RIT2* 5′-upstream fragments, −1290 to +76 bp (*RIT2*-1290/luciferase) and −374 to +76 bp (*RIT2*-374/luciferase), that were amplified by PCR using a P1 clone as the template and the primers listed in Appendix 1. Both fragments were ligated into the SmaI site of pGL2-Basic, which contains the firefly luciferase gene (Promega, Madison, WI), and confirmed by sequencing.

To construct human POU4 expression vectors, full-coding cDNAs of POU4F1, POU4F2, and POU4F3 were generated by reverse transcription (RT)-PCR using total RNA from SK-N-MC human neuroblastoma cells by oligo(dT) priming and PCR amplification (Appendix 1). Forward primers for all POU4 factors included an EcoRI site. Reverse primers included a HindIII site for POU4F1 and POU4F2, and a BamHI site for POU4F3. The cDNA fragments were inserted into the EcoRI/HindIII or EcoRI/BamHI sites in the pcDNA3.1/Myc-His(-) B vector (Invitrogen, Carlsbad, CA), and confirmed by sequencing. A human ISL1 expression vector was constructed in the same manner except that the ISL1 cDNA was made using retinal RNA from human donor eyes (National Disease Research Interchange, NDRI). The forward primer included an EcoRI site, and the reverse primer included a HindIII site (Appendix 1).

### Cell culture and reverse transcription polymerase chain reaction

Human neuroblastoma cell lines, SK-N-MC [37], SK-N-AS [38], and SK-N-DZ [39], and the human embryonic kidney line HEK293 [40] (American Type Culture Collection [ATCC], Manassas, VA), were cultured in Dulbecco's Modified Eagle Medium (Invitrogen) containing 10% fetal bovine serum supplemented with penicillin and streptomycin (Invitrogen) at 37 °C with 5% CO_2_ and 95% air. Total RNAs were extracted using TRIzol (Invitrogen) from subconfluent cell cultures or human retina (NDRI), and human brain RNA was purchased (Clontech, Mountain View, CA). First-strand cDNAs were synthesized from 1 μg of total RNAs with oligo(dT) primer and SuperScript III reverse transcriptase (Invitrogen). A 381 bp *RIT2* fragment was amplified by 35 cycles of PCR using a forward primer in exon 4 and a reverse primer in exon 5, and ribosomal protein *S16* was used as the control (Appendix 1).

### Laser capture microdissection and reverse transcription quantitative polymerase chain reaction

All mice were treated in accordance with the Guide for the Care and Use of Laboratory Animals and the Animal Welfare Act as well as the guidelines of the Institutional Animal Care and Use Committee at Johns Hopkins University. Tissues of individual retinal layers of adult C57BL/6J mice were collected by laser capture microdissection (LCM) as previously described [41-43]. Briefly, after the cornea and lens were removed, the eyecups were immersed in 10%, 15%, and 25% sucrose for 30 min each, and frozen in optimal cutting temperature compound (OCT) with 25% sucrose. Sections were cut at 7 μm, mounted onto slides with charged PEN-foil membranes (Leica Microsystems, Deerfield, IL), fixed in 70% ice-cold ethanol for 30 s, stained with hematoxylin for 10 s, and dehydrated in 70% and 100% ethanol for 1 min each. Tissues of the outer nuclear layer (ONL), inner nuclear layer (INL), and ganglion cell layer (GCL) were isolated separately using an LMD6000 laser capture microdissection microscope (Leica Microsystems). Total RNAs were extracted from each layer using an RNeasy Micro kit (Qiagen, Valencia, CA), and first-strand cDNAs were synthesized by random hexamer priming with SuperScript III reverse transcriptase (Invitrogen). Quantitative PCR (qPCR) analyses were performed with primers listed in Appendix 1 using iQ SYBR Green SuperMix on an iQ5 Real-Time PCR Detection System (Bio-Rad, Hercules, CA). A relative amount of cDNA of each gene was normalized to that of control *Gapdh*.

### Immunohistochemistry of retinal flat mounts and eye sections

Protein expression of RIT2 and POU4F2 was analyzed by immunohistochemistry of retinal flat mounts and eye sections using 7- to 9-week-old C57BL/6J mice as well as mouse eye sections at embryonic day 15.5 (E15.5) and postnatal day 0 (P0) following published protocols [35,44]. Briefly, after the cornea and lens were removed, the eyecups were fixed in ice-cold 4% paraformaldehyde in phosphate-buffered saline (PBS; 10 mM Na_2_HPO_4_, 2 mM KH_2_PO_4_, 137 mM NaCl, 2.7 mM KCl, pH 7.4) for 1 h, and washed in 0.3% Triton X-100 in PBS (0.3% PBST). The retinas were dissected, cut from the periphery toward the optic disc, and placed on a slide with the GCL facing up. The retinal flat mounts were blocked with 10% normal donkey serum in 0.1% PBST for 1 h, and then incubated with a primary antibody, anti-RIT2 antibody (1:200; PA1–25559, rabbit polyclonal, Thermo Scientific, Rockford, IL) or anti-POU4F2 antibody (1:500; sc-6026, goat polyclonal, Santa Cruz Biotechnology, Santa Cruz, CA), in 5% normal donkey serum in 0.1% PBST at 4 °C for 2 days. After several washes with 0.1% PBST, the flat mounts were incubated with a secondary antibody, Alexa Fluor 488 donkey anti-rabbit immunoglobulin (IgG; 1:1,000; A-21206, Invitrogen) for RIT2 or Alexa Fluor 647 donkey anti-goat IgG (1:1,000; A-21447, Invitrogen) for POU4F2, for 2 h at room temperature. The flat mounts were washed with 0.1% PBST, mounted in VECTASHIELD HardSet Mounting Medium with 4', 6-diamidino-2-phenylindole (H-1500, Vector Laboratories, Burlingame, CA), and examined on an LSM 510 inverted laser scanning confocal microscope (Carl Zeiss, Thornwood, NY). For the initial analyses of RIT2 expression by immunohistochemistry of the retinal sections, the mouse eyes were fixed in ice-cold 4% paraformaldehyde in PBS for 1 h, washed in 0.3% PBST, cryoprotected in 20% sucrose in PBS at 4 °C overnight, embedded in OCT Tissue-Tek (Ted Pella, Redding, CA), and cut at 12 μm on a cryostat. The eye sections were incubated with the same primary and secondary antibodies as used for the flat mounts, and examined in the same manner as described above.

For immunohistochemistry to further analyze cell-type-specific RIT2 expression in the INL, eye tissues were processed using shorter fixation as previously described [43,45]. Briefly, after the anterior segment of the eye was removed, the eyecups were fixed for 25 min in cold 4% paraformaldehyde in 0.1 M phosphate buffer, pH 7.4, and subjected to an increasing gradient of sucrose (6.75%, 12.5%, and 25%) in phosphate buffer. The eyecups were then immersed for 1 h in a 2:1 ratio of 25% sucrose in 0.1 M phosphate buffer and OCT Tissue-Tek (Ted Pella), and snap frozen on dry ice in isopentane. Cryosections were cut at 8–10 μm, simultaneously blocked and permeabilized in 2% normal donkey serum in 0.1% PBST, and incubated overnight with a primary antibody in the blocking solution. Primary antibodies used were anti-RIT2 (1:200; PA1–25559, Thermo Scientific), anti-PAX6 (1:500; Developmental Studies Hybridoma Bank, DSHB, mouse monoclonal, University of Iowa, Iowa City, IA), anti-calbindin D28 (1:1,000; CL300, mouse monoclonal, Sigma, St. Louis, MO), anti-vimentin (1:100; DSHB), anti-LIM1 (1:50; DSHB), and anti-visual system homeobox 2 (VSX2; 1:1,000; Ab9016, sheep polyclonal, Millipore, Billerica, MA). Secondary antibodies were anti-mouse, anti-sheep, and anti-rabbit IgG (heavy and light chains) coupled to Alexa Fluor 488 or 647 (1:1,000; Invitrogen). Hoechst 3342 (Molecular Probes, Invitrogen) was used at 10 μg/ml to visualize cell nuclei. Serial sections processed similarly but without a primary antibody were used as the control for the background. Images of retinal sections were acquired with Zeiss laser scanning confocal microscopes LSM 510 and LSM 710, and images were adjusted for brightness and contrast using ImageJ (NIH, Bethesda, MD) or Photoshop (Adobe, San Jose, CA).

### Western blotting

Protein lysates were prepared from the mouse retinas at P2, P5, P15, and adult (8 weeks old) and used for western blot analysis. Briefly, the retina was harvested from two eyecups at each time point, rinsed with ice-cold PBS, and lysed by sonication in 200 μl radio immunoprecipitation assay (RIPA) lysis buffer (R0278, Sigma) on ice. The lysed retinal tissues were mixed gently, kept on ice for 15 min, and centrifuged at approximately 14,000 ×*g* for 15 min to pellet the debris. The supernatant was transferred to a fresh tube, and the protein concentration was measured using DC Protein Assay (500–0113, 500–0114, and 500–0115, Bio-Rad). After heating at 99 °C for 5 min, 30 μg of protein lysates for each sample were resolved with sodium dodecyl sulfate–polyacrylamide gel electrophoresis and transferred to a nitrocellulose membrane. The blotted membrane was then blocked in TBST (10 mM Tris-Cl [pH 7.5], 100 mM NaCl, 0.1% Tween-20) containing 5% non-fat dry milk at room temperature for 1 h and probed with anti-RIT2 antibody PA1–25559 (1:1,000; Thermo Scientific) or anti-α tubulin antibody (1:500; T9026, mouse monoclonal, Sigma) in TBST containing 5% non-fat dry milk at 4 °C overnight. After washing with TBST three times, the membrane was incubated with horseradish peroxidase–conjugated anti-rabbit IgG and anti-mouse IgG antibodies (1:10,000; 7074 and 7076, Cell Signaling Technology, Danvers, MA) for detecting RIT2 and α tubulin, respectively, at room temperature for 1 h. After washing with TBST, protein signals were visualized with chemiluminescence using SuperSignal West Femto Maximum Sensitivity Substrate (PI34095, Thermo Scientific) with BioMax MR X-ray films (8941114, Kodak, Rochester, NY), and the signal intensity was analyzed with ImageJ (NIH).

### Bioinformatic analysis of *RIT2* upstream region

MatInspector [46] was used in combination with the Matrix Family Library database (Genomatix, Munich, Germany), and more recently with the MatBase database (Genomatix), to analyze the upstream region of *RIT2*. The matrix-based pattern matching program Patser [47,48] (Regulatory Sequence Analysis Tools) [49] was used with two published position-specific scoring matrices, Matrix 1 for POU4F1/F2 [50] and Matrix 2 for POU4F2 [27], to scan for the presence of potential binding sites for POU4 proteins in the *RIT2* upstream region from −1290 to +76 bp.

### Electrophoretic mobility shift assays

Electrophoretic mobility shift assays (EMSAs) were performed as previously described [51,52]. Briefly, proteins to be tested, POU4F1-s (short), POU4F1-l (long), POU4F2-s, POU4F2-l, and POU4F3, as well as control luciferase protein, were generated in vitro using the TnT T7 Quick Coupled Transcription-Translation System (Promega). Synthesis of the appropriate protein was confirmed in a separate reaction by ^35^S-methionine labeling followed by sodium dodecyl sulfate–polyacrylamide gel electrophoresis separation and autoradiography. Six oligonucleotide probes (Probes 1-6), which were 24 bp long and contained each predicted POU4 binding site in the middle, were labeled by fill-in reaction as previously described [53]. Briefly, oligonucleotide pairs complementary to each other were annealed to create overhangs, mixed with [α-^32^P] deoxycytidine triphosphate and a mixture of non-radioactive deoxyadenosine triphosphate, deoxythymidine triphosphate, and deoxyguanosine triphosphate, and the recessed ends were extended using Klenow fragment. Mutated probes were made for Sites 1, 2, and 6 (Probes 1m, 2 m, and 6 m, respectively) by introducing a mutation (TTAA to GGCC). The labeled probe (about 30,000 cpm) was incubated with 3 μl of in vitro generated protein in binding solution (10 mM Tris-Cl, pH 7.9, 100 mM KCl, 5 mM MgCl_2_, 1 mM EDTA, 1 mM dithiothreitol, 0.5 mM phenylmethylsulfonyl fluoride, 5% glycerol) containing 1 μg poly(dI-dC) on ice for 30 min and analyzed on a 5% polyacrylamide gel. For cold oligomer competition, labeled Probe 2 was mixed with 1-, 10-, 100-, and 1,000-fold molar excess of unlabeled competitor oligonucleotides, and then added to the binding solution containing the POU4F1-s protein.

### Transient transfection

In all experiments, cells were transfected 24–48 h after plating using Lipofectamine LTX (Invitrogen). For transfection with a *RIT2* promoter-luciferase construct alone, neuroblastoma cells in a 60-mm dish were transfected with 3 μg of a luciferase construct (*RIT2*-1290/luciferase, *RIT2*-374/luciferase, or empty pGL2-Basic) and 0.5 μg of pCMV*-lacZ* as the internal control. Cell lysates were prepared 48–60 h after transfection using Reporter Lysis Buffer (Promega), and luciferase and β-galactosidase activities were measured as previously described [53]. Luciferase activity was normalized with β-galactosidase activity, and relative luciferase activity was calculated as the ratio of the normalized luciferase activity with *RIT2* promoter constructs to that with pGL2-Basic. For cotransfection with a *RIT2* promoter-luciferase construct and POU4 and/or ISL1 expression vectors, SK-N-AS cells in 12-well plates were transfected with 0.2 μg of a firefly luciferase construct, 0.4 μg of each pcDNA3.1 expression vector (either with cDNA or no insert), and 0.1 ng of pRL-CMV containing the *Renilla* luciferase gene (Promega). The total amount of the expression vectors was adjusted to 0.8 μg with empty pcDNA3.1 in all experiments. Cell lysates in Passive Lysis Buffer (Promega) were analyzed using the Dual-Luciferase Reporter System (Promega). Firefly luciferase activity was normalized with *Renilla* luciferase activity, and relative luciferase activity was calculated as the ratio of the normalized luciferase activity with pcDNA3.1 containing cDNA to that with empty pcDNA3.1. Three to four independent transfections were performed in duplicate each time. Statistical analysis was performed using the Student *t* test to compare relative luciferase activity with a POU4 factor and ISL1 to that with a POU4 factor alone.

## Results

### The 5′-upstream region of human *RIT2* drives expression in neuronal cells

As an initial step for identifying the *RIT2* promoter, we searched genomic databases at the National Center for Biotechnology Information (NCBI) and the University of California Santa Cruz (UCSC) genome browser. These analyses revealed that the human and mouse genes encoding RIT2 are unusually large; they contain only five exons, with the coding region totaling 651 bp, but span 300 kb due to their extensive introns. In contrast, the genes encoding RIT1, a homolog of RIT2, are small and extend only a little over 10 kb (Appendix 2). Although the presence of such large introns in *RIT2* raises the possibility that intronic sequences could contribute to its neuron-specific expression pattern, we decided initially to focus on *RIT2*’s immediate upstream region. We first determined the transcription start site for *RIT2* by primer extension using total RNA from human retinas. The strongest signal was seen at 179 bp upstream of the initiation ATG (data not shown). This position is 5 bp upstream of the messenger RNA (mRNA) 5′-end annotated in RefSeq (GenBank: NM_002930). Sequence alignment of the 5′-flanking region of human and mouse *Rit2* revealed many blocks of conserved sequences within the 1 kb upstream from the transcription start site and in the 5′-untranslated region. Based on the concept that the conserved regions are more likely to contain functional regulatory elements, we chose the 1.3 kb upstream containing the conserved 1 kb region as a tentative promoter, and constructed two human *RIT2* promoter-luciferase reporter vectors, *RIT2* −1290 to +76 bp (*RIT2*-1290/luciferase) and −374 to +76 bp (*RIT2*-374/luciferase), by PCR. As the template for PCR, we used a P1 genomic clone for human *RIT2*, instead of human genomic DNA, because *RIT2* genomic clones had already been obtained earlier by screening a human genomic P1 library, and therefore were available in the laboratory.

**Figure 1:**
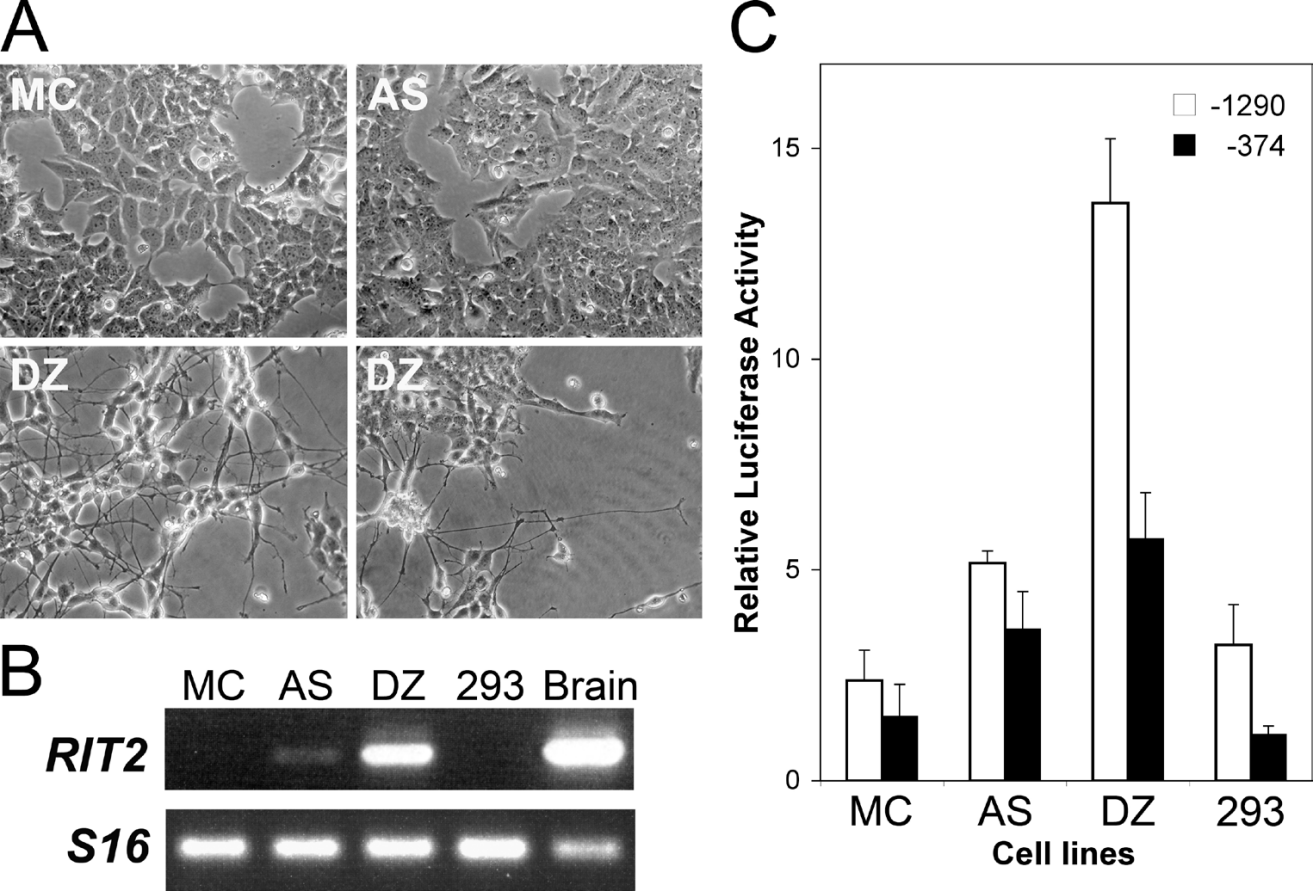
The 5′-upstream region of human *RIT2* drives expression in neuronal cells. **A**: This panel shows morphology of human neuroblastoma cells. Cells were grown as described in Methods and photographed with an inverted microscope. Cell lines used are SK-N-MC (labeled as MC), SK-N-AS (AS), and SK-N-DZ (DZ). **B**: This panel shows endogenous *RIT2* expression. Total RNA was extracted from subconfluent cell cultures and analyzed by reverse transcription-polymerase chain reaction. *S16* expression was used as a normalization control. Samples are SK-N-MC (labeled as MC), SK-N-AS (AS), SK-N-DZ (DZ), HEK293 (293), and human brain RNA as the positive control (brain). **C**: *RIT2* promoter activity was analyzed by transfection. A luciferase construct containing a 5′-upstream fragment of *RIT2*, either –1290 to +76 (labeled as −1290, open column) or –374 to +76 bp (−374, solid column), or empty pGL2-Basic vector as the background control was transiently transfected into the indicated cells together with pCMV-*lacZ* as internal control for transfection efficiency. Luciferase activities were normalized with β-galactosidase activities, and relative luciferase activities were calculated as the ratio of the normalized luciferase activity with constructs containing *RIT2* upstream fragments to that with empty pGL2-Basic. The values represent the means and standard deviation (error bar).

Next, we used transient transfection assays to identify promoter activity of the 5′-upstream region of human *RIT2*. For these studies, we sought a cell line expressing *RIT2*, since such a cell line would more likely mimic the in vivo regulatory milieu compared to non-expressing cells. We screened three human neuroblastoma cell lines, SK-N-MC, SK-N-AS, and SK-N-DZ [37-39], and the transformed embryonic kidney line HEK293 [40] for *RIT2* expression by RT-PCR using the human brain as the positive control. Morphologically, SK-N-MC (labeled as MC) and SK-N-AS (AS) did not show notable neurite outgrowth, whereas SK-N-DZ (DZ) extended long processes and formed intricate networks of neurites (Figure 1A). Interestingly, given that RIT2 has been implicated in neurite outgrowth [10], the expression levels of endogenous *RIT2* generally correlated with the degree of neurite outgrowth, with SK-N-DZ expressing *RIT2* at the highest level (Figure 1B). *RIT2* expression was undetectable in HEK293. Using the three neuroblastoma and HEK293 cell lines, we performed transient transfection with *RIT2*-1290/luciferase and *RIT2*-374/luciferase. Promoter activity of *RIT2*-1290/luciferase overall correlated with the level of endogenous *RIT2* expression, with the highest activity observed in SK-N-DZ (Figure 1C). Promoter activity of *RIT2*-374/luciferase was approximately 40% of that of *RIT2*-1290/luciferase in SK-N-DZ, but the difference in activity between the cell lines was not as prominent as seen with the longer −1290 fragment. These results suggest that the *RIT2* −1290 to +76 bp region has promoter activity that drives expression preferentially in differentiated neuronal cells, and that at least some of the regulatory elements responsible for neuronal expression are located in the region between –1290 and –374 bp.

### *Rit2* is expressed in the ganglion cell layer and inner nuclear layer of the mouse retina

**Figure 2:**
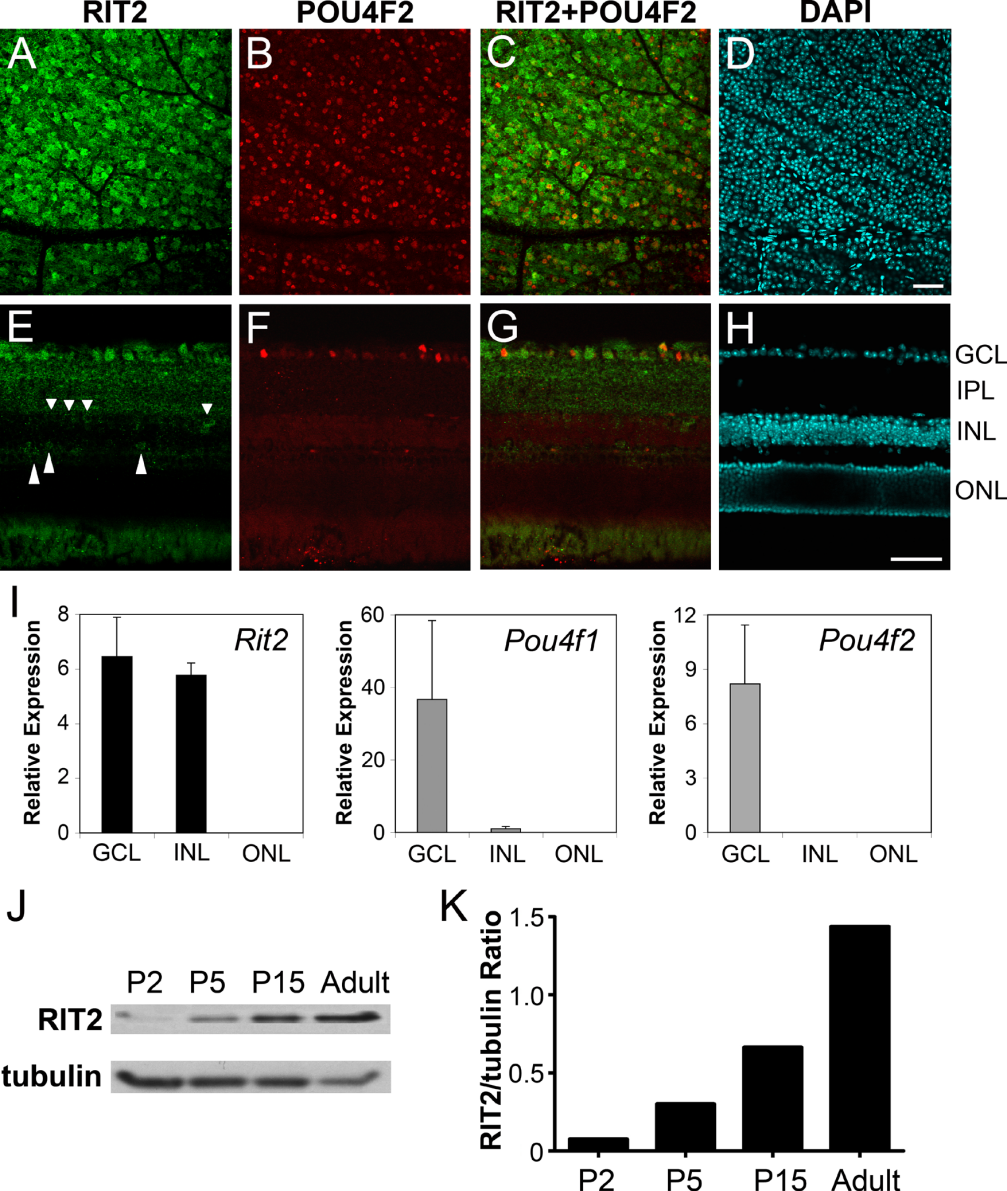
Expression of *Rit2* mRNA and protein in the mouse retina. The top panels show immunohistochemistry of retinal flat-mounts (**A**-**D**) and retinal sections (**E**-**H**). Images are staining with anti-RIT2 antibody (**A**, **E**), anti-POU4F2 antibody (**B**, **F**), merged image of RIT2 and POU4F2 (**C**, **G**), and nuclear staining by 4', 6-diamidino-2-phenylindole (DAPI; **D**, **H**). RIT2-positive cells are more numerous than POU4F2-positive RGCs. **E**: RIT2 protein is detected in the ganglion cell layer (GCL), inner plexiform layer (IPL), and cells in the inner nuclear layer (INL). Short arrowheads point to RIT2-positive cells located at the inner border of the INL; long arrowheads point to RIT2-positive cells at the outer aspect of the INL. Scale bar: 50 μm. **I**: This panel shows *Rit2* mRNA expression. Laser capture microdissection was used to collect tissues of GCL, INL, and outer nuclear layer (ONL) from mouse retinal sections, and RNA of each layer was analyzed by reverse transcription-quantitative polymerase chain reaction. The mRNA level of *Rit2*, *Pou4f1*, and *Pou4f2* was normalized by that of *Gapdh*. **J**: RIT2 protein levels were analyzed at different ages. Protein lysates were prepared from mouse retinas at postnatal day 2 (P2), P5, P15, and adult (8 weeks old), and analyzed by western blotting with anti-RIT2 antibody and anti-α tubulin antibody for control. Protein signals were visualized by chemiluminescence with X-ray films. **K**: This panel shows relative quantification of RIT2 protein levels. Western blot signals on the X-ray films were scanned and analyzed with ImageJ software. The signal intensity of RIT2 was normalized by that of α tubulin and presented as the RIT2/tubulin ratio. RIT2 protein levels increased from P2 to adults by more than 15 fold.

Because of our interest in retinal biology, we next aimed to clarify the expression pattern of *Rit2* in the retina. Although we had previously shown by in situ hybridization that *Rit2* mRNA is present in RGCs [5], we wanted to examine RIT2 expression at the protein level by immunostaining retinal flat mounts and sections with a focus on RGCs. We analyzed the expression of RIT2 and POU4F2 proteins, with the latter serving as an RGC marker. In retinal flat mounts (Figure 2A–D), RIT2 was detected in the cell body with stronger staining in cell membranes, and RIT2-positive cells were more numerous than POU4F2-positive cells. Because approximately 70% of RGCs express POU4F2, and the GCL contains displaced amacrine cells, the staining pattern suggests that RIT2 is expressed in both RGCs and amacrine cells. Immunohistochemistry of retinal sections (Figure 2E–H) showed RIT2 protein in all GCL cells, and this distribution was broader than that of POU4F2, confirming the results of the flat mounts. In addition, RIT2 protein was also detected in the inner plexiform layer (IPL) and in cells located at the inner and outer borders of the INL (Figure 2E; short and long arrowheads, respectively). We also analyzed RIT2 expression in the mouse embryonic retina by immunohistochemistry. Weak staining of the RIT2 protein was observed in the GCL and inner neuroblastic layer (NBL) at E15.5, and RIT2 expression significantly increased from E15.5 to newborn to adults (Appendix 3).

We further analyzed *Rit2* retinal expression using LCM. The GCL, INL, and ONL fractions were collected by LCM of mouse retinal sections, and the resulting samples were analyzed by RT-qPCR to measure the mRNA levels of *Rit2*, *Pou4f1*, *Pou4f2*, and *Gapdh*. Consistent with the immunohistochemistry data, *Rit2* was highly expressed in the GCL and INL, but *Rit2* mRNA was not detected in the ONL sample (Figure 2I). *Pou4f1* and *Pou4f2* mRNAs were detected predominantly and exclusively in the GCL, which is consistent with published results [26,27,29,34]. Interestingly, *Pou4f1* mRNA was also detected in the INL, albeit at a low level. This observed *Pou4f1* expression in the INL is unlikely due to contamination of the samples because *Pou4f2* mRNA was not similarly detected in the INL (Figure 2I). We also analyzed RIT2 protein levels in the mouse retina at different ages by western blotting. RIT2 protein levels increased from P2 to adults by more than 15-fold (Figure 2J,K).

### RIT2 protein is detected in all neuronal cell types in the inner nuclear layer

**Figure 3:**
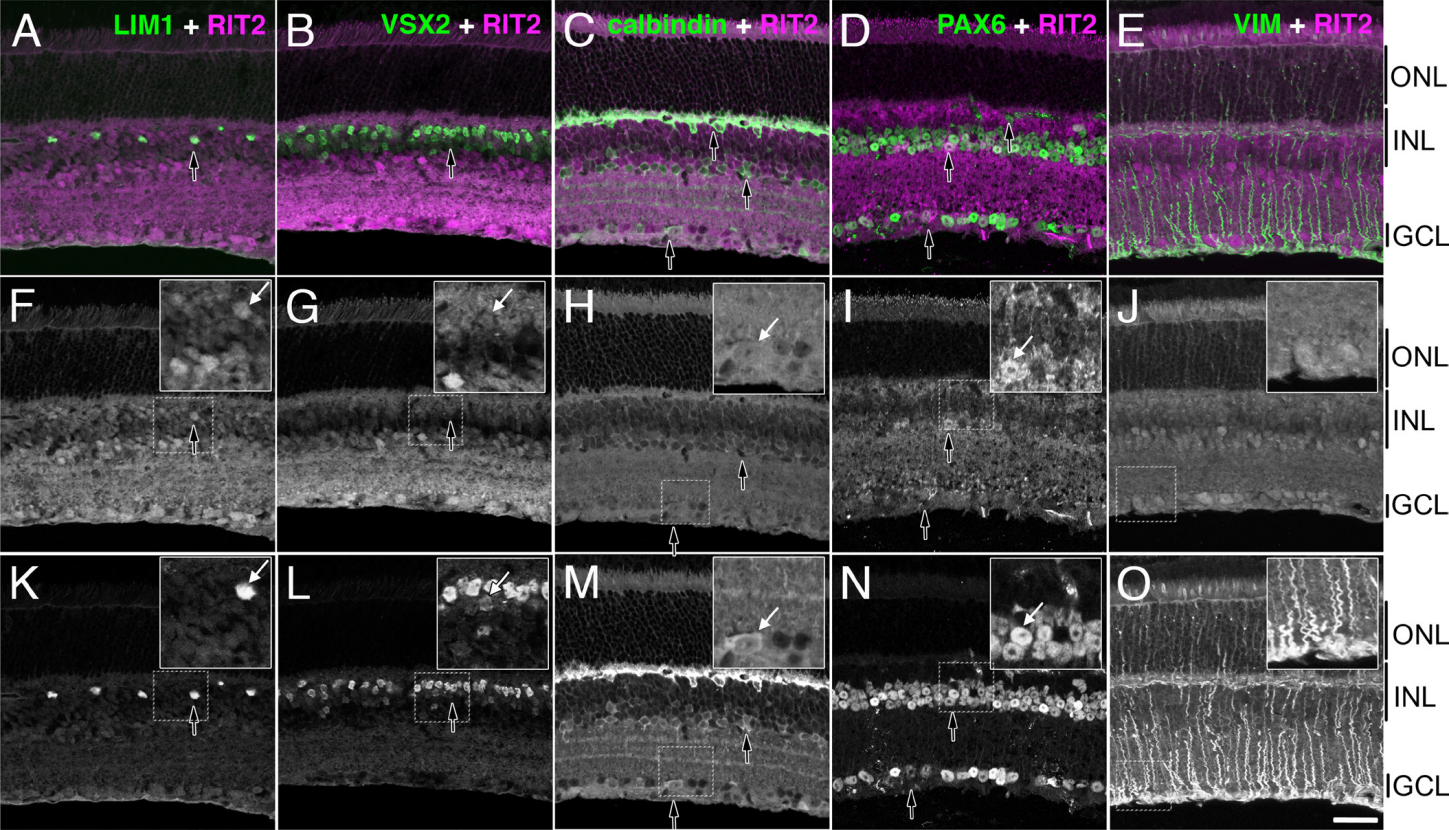
Double-label immunohistochemistry reveals RIT2 expression in neuronal cells in the inner nuclear layer (INL). Images are double labeling of RIT2 with the horizontal cell marker LIM1 (**A**, **F**, **K**), the bipolar marker VSX2 (**B**, **G**, **L**), markers for a mixed population of horizontal, amacrine, and ganglion cells calbindin D28 (**C**, **H**, **M**) and PAX6 (**D**, **I**, **N**). Non-neuronal Müller glia was detected with an antibody against vimentin (**E**, **J**, **O**). Color images in the top panels represent merged images of double labeling for RIT2 and one of the cell-type-specific markers (**A–E**). Black and white images in the middle and bottom panels show single channel images of RIT2 (**F–J**) and the cell-type-specific markers (**K**–**O**), respectively. Arrows indicate representative cells that exhibit colocalization of RIT2 and each cell-type-specific marker, which is shown in a higher magnification in the insets. Scale bar: 20 μm. GCL, ganglion cell layer; INL, inner nuclear layer; ONL, outer nuclear layer.

During the initial analyses of RIT2 expression with a focus on RGCs, we also detected RIT2 expression in cells of the INL by immunohistochemistry of the retinal sections. To identify the cell types that express RIT2 in the INL, we performed double-labeling immunohistochemistry (Figure 3) with markers preferentially expressed in horizontal cells (LIM1), bipolar cells (VSX2), and Müller glia (vimentin, labeled as VIM) as well as two markers that stain horizontal, amacrine, and ganglion cells (calbindin and PAX6). We observed colocalization between RIT2 and each of the neuronal cell markers, but failed to see overlap with vimentin. Nuclear localized LIM1 expression had a near perfect overlap with RIT2 in cells located at the outer aspect of the INL where horizontal cells are present (Figure 3A,F,K). Similar colocalization was observed between VSX2 and RIT2 (Figure 3B,G,L). Antibodies against calbindin D28 labeled horizontal cells, including their dendrites, as well as a subset of amacrine cells and cells in the GCL. Although calbindin D28 generally has a stronger and more discrete signal and a better separation of sublaminae of the IPL, RIT2 and calbindin D28 exhibited a high degree of overlap, particularly regarding horizontal cell bodies and their dendritic arbors (Figure 3C,H,M). PAX6, although often used as a marker for amacrine and ganglion cells, is also expressed in horizontal cells; however, the spatial separation of these cells in the retina makes it easy to distinguish them (Figure 3D,I,N). Therefore, we confirmed the identity of these cells by the association of RIT2 and PAX6. The only marker that failed to show a close association with RIT2 was the Müller radial glial marker vimentin (Figure 3E,J,O). The thin thread-like projections spanning the retina were intensely labeled with vimentin but not with RIT2. Taken together, it appears that all neuronal cell types in the INL express RIT2, while Müller glia does not. In addition to cells in the INL, we also observed RIT2 positive staining, although weaker, in the inner segments of photoreceptors. This observation conflicts with the LCM RT-qPCR results, which showed no detectable *Rit2* expression in the ONL (Figure 2I), and remains to be resolved.

### Bioinformatic prediction of POU4 protein binding sites

**Figure 4:**
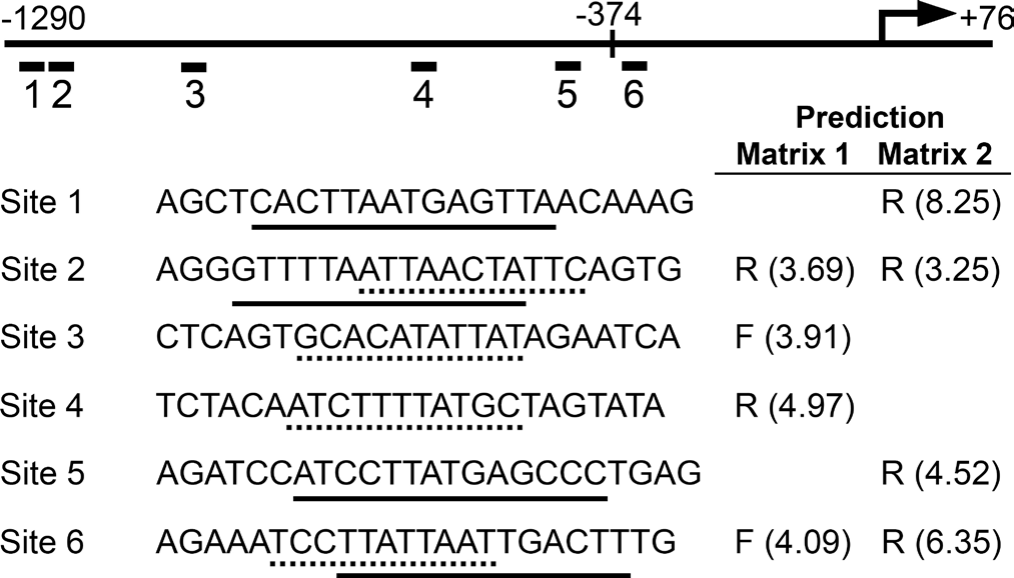
Bioinformatic prediction of POU4 protein binding sites. Nucleotide sequence of the *RIT2* upstream region from –1290 to +76 bp was scanned to search potential POU4 protein binding sites using the Patser program with two position-specific scoring matrices, POU4F1/F2 matrix (Matrix 1) [50] and POU4F2 matrix (Matrix 2) [27]. The transcription start site (+1) is indicated with an angled arrow, and the position of –374 bp is marked with a vertical line. Using 3.0 as a cut-off score, four sites were predicted with each matrix, and two sites (Sites 2 and 6) were predicted with both matrices. The positions of the predicted sites (Sites 1–6) are indicated by short lines with numbers under the *RIT2* –1290 to +76 bp genomic segment. The sequences of oligonucleotides containing the predicted sites in the middle for EMSA are shown. Core binding motifs predicted by Matrices 1 and 2 are marked with dotted and solid underlines, respectively (see also Appendix 4). Strands of the predicted sites are also shown as F (forward) or R (reverse) with scores in parentheses.

To identify candidate regulatory elements within the *RIT2* 1.3 kb promoter region, we repeatedly searched for the presence of predicted transcription factor binding sites using MatInspector and found a number of consensus binding sites, including multiple AP1, four E-box, multiple POU domain factor including seven POU4F, a CREB1, and an SP1 sites. Based on the essential role of POU4 factors in the development and survival of sensory ganglion neurons that also express *RIT2*, we hypothesized that POU4 factors might regulate *RIT2* in RGCs. We therefore searched possible binding sites for POU4 proteins in the *RIT2* upstream region in further detail using a different program, Patser [49], with the published position-specific scoring matrices for POU4F1/F2 (Matrix 1) [50] and POU4F2 (Matrix 2) [27], of which the consensus elements are gcATAATTAAT and a/gCTCATTAAt/c, respectively. Although the former consensus site was shown to have 1,000-fold higher affinity than the latter consensus site [50], we used both matrices for comparison in the case of our native *RIT2* promoter elements. Patser predicted 15 and 17 sites using Matrices 1 and 2, with scores ranging from 4.97 to 0.07 (highest possible score 12.195) and 8.25 to 0.05 (highest possible score 12.542), respectively. Using a semiarbitrary cut-off score of 3.0, four sites were predicted with each Matrix, and Sites 2 and 6 were predicted with both matrices, resulting in a total of six predicted sites (Sites 1–6; Figure 4 and Appendix 4).

### POU4 proteins bind to the predicted sites

**Figure 5:**
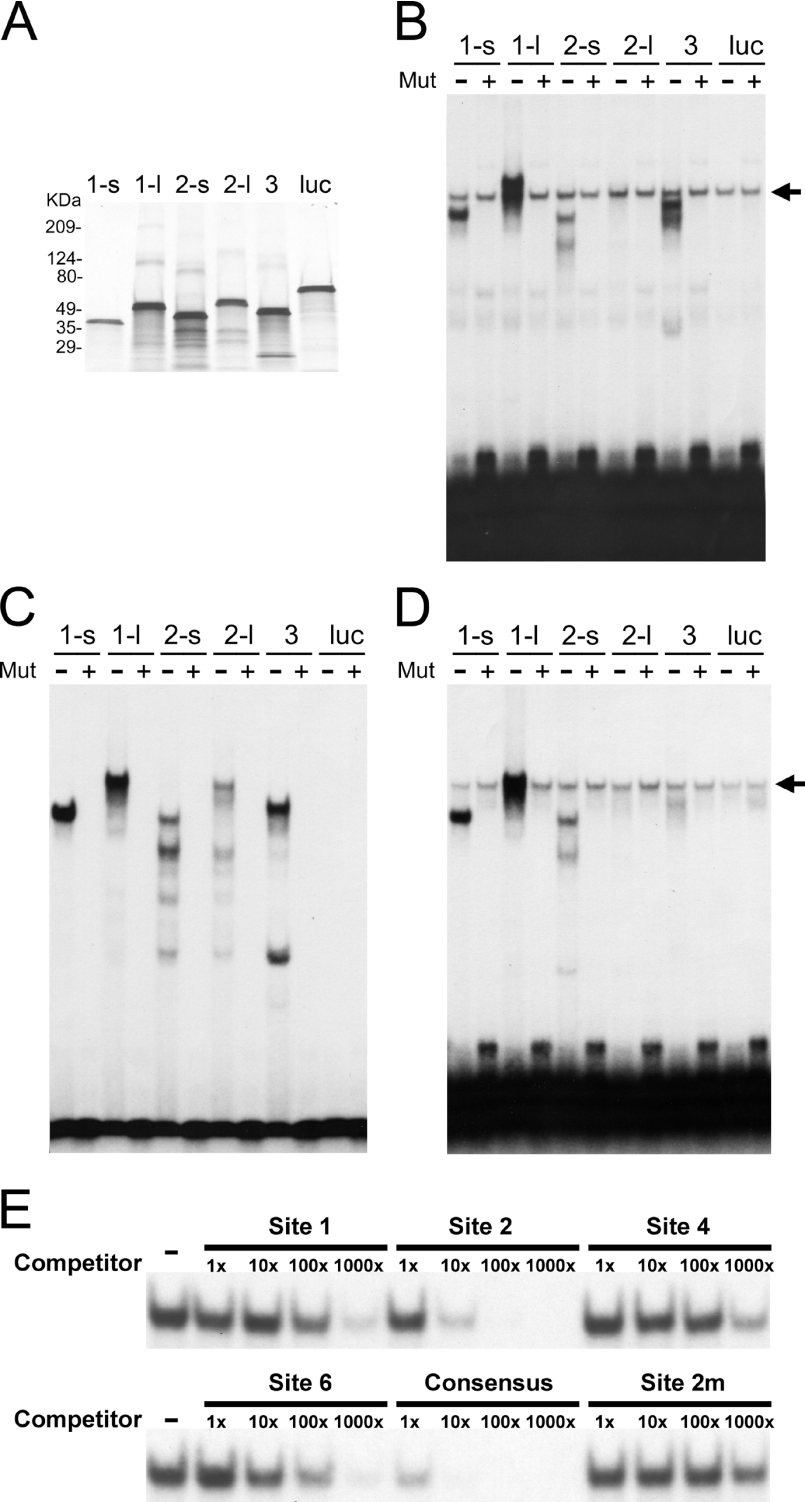
POU4 proteins bind to the predicted sites in a sequence-specific manner. **A**: This panel shows POU4 proteins produced by in vitro transcription and translation. The efficiency of protein synthesis was tested by labeling proteins with ^35^S-methionine during translation, followed by sodium dodecyl sulfate-polyacrylamide gel electrophoresis (SDS–PAGE) fractionation and autoradiography. Proteins were POU4F1-s (labeled as 1-s), POU4F1-l (1-l), POU4F2-s (2-s), POU4F2-l (2-l), POU4F3 (3), and luciferase (luc). **B**: Electrophoretic mobility shift assay (EMSA) was performed with probes containing Site 1. The sequence of Probe 1, which is 24 bp annealed oligonucleotides containing Site 1 in the middle, is shown in Figure 4. Mutated Probe 1 contains a mutation (TTAA to GGCC) in the core binding sequence of Site 1. Either ^32^P-labeled wild-type (labeled as Mut -) or mutated (Mut +) Probe 1 was incubated with 3 μl of each in vitro translated protein. A non-specific band seen in all lanes is indicated by arrow. Proteins were same as in A. **C**: This panel shows EMSA with probes containing Site 2. The experimental design and result presentation are the same as in B, except that Probe 2 containing Site 2 was used, and a non-specific band was not observed. **D**: This panel shows EMSA with probes containing Site 6. The experimental design and result presentation are the same as in B, except that Probe 6 containing Site 6 was used. **E**: EMSA was also performed for cold oligomer competition. ^32^P-labeled Probe 2 was mixed with 1x, 10x, 100x, and 1000×(fold) molar excess of unlabeled competitors, and then incubated with POU4F1-s protein. Competitors used were oligonucleotides containing Sites 1, 2, 4, 6, mutated 2 (2m), and the reported POU4F1 consensus site. Binding with no competitor (labeled as Competitor -) is included as control.

To experimentally validate Sites 1–6, we performed EMSAs with proteins made by in vitro translation for all POU4 factors and their isoforms [34,54]. The production of five POU4 (POU4F1-s [short isoform], POU4F1-l [long], POU4F2-s, POU4F2-l, and POU4F3) and control luciferase proteins was confirmed by ^35^S-methionine labeling, and clear major bands of the expected size were obtained for all (Figure 5A). First, we performed EMSAs using Probes 1–6, corresponding to Sites 1–6 (Figure 4), with unlabeled POU4 and luciferase proteins. Probe 2 yielded strong shifted bands with all POU4 proteins within an hour of autoradiography (Figure 5C), whereas 2–3 days of exposure were required to obtain clear bands with Probes 1 and 6 (Figure 5B, D), and even after exposure for 7 days, Probes 3, 4, and 5 did not show detectable shifts (data not shown). Next, we tested the sequence-specificity of binding using wild-type (Probes 1, 2, and 6) and mutated (TTAA to GGCC; Probes 1m, 2m, and 6m) probes with the POU4 and luciferase proteins. All shifted bands were lost with the mutated probes (Figure 5B–D). To compare the relative binding strength of these sites, we employed cold oligomer competition. Binding of ^32^P-labeled Probe 2 to POU4F1-s protein was competed with unlabeled “competitor” oligonucleotides containing Sites 1, 2, 4, 6, and 2m as well as the reported POU4F1 consensus site [50]. The strongest competition, i.e., competition with the lowest amount, was observed with the POU4F1 consensus site (Figure 5E). The competition with Site 2 was 10-fold weaker than that with the consensus site, but 10-fold- to 100-fold stronger than that with Sites 1 and 6. In contrast, Site 4 competed only marginally, and Site 2m hardly competed (Figure 5E). In summary, of the six predicted sites, three were bound by POU4 proteins under the conditions used, and the relative binding strength was approximately 1,000, 100, 1–10, and 1–10 for the POU4F1 consensus site, Sites 2, 6, and 1, respectively.

### Different POU4 proteins show distinct activities in modulating the *RIT2* promoter

**Figure 6:**
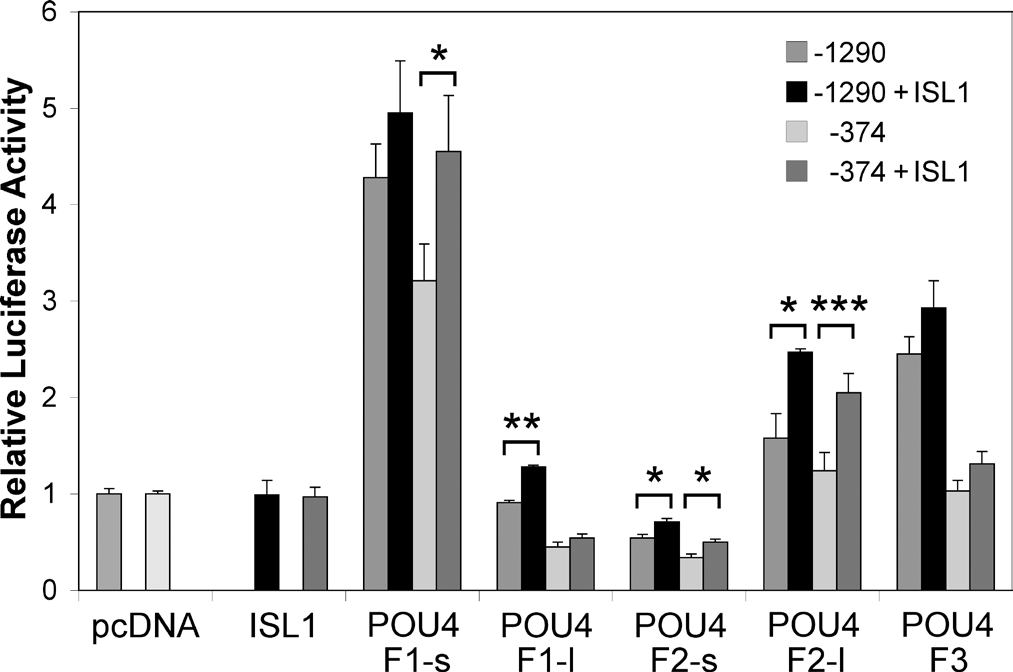
POU4 factors modulate the *RIT2* promoter. A firefly luciferase construct containing a fragment *RIT2* –1290 to +76 bp (labeled as –1290) or –374 to +76 bp (–374) was cotransfected into SK-N-AS cells with pcDNA3.1 expression vectors containing cDNA of the indicated transcription factors, together with pRL-CMV containing the *Renilla* luciferase gene for normalization. Expression vectors tested were for POU4 factor (labeled as POU4F1-s, POU4F1-l, POU4F2-s, POU4F2-l, and POU4F3 on the x-axis), ISL1, or no insert as control (labeled as pcDNA). Relative luciferase activity was calculated as the ratio of the normalized luciferase activity with POU4 and/or ISL1 expression vectors to that with empty pcDNA3.1. The values represent the mean and SEM (error bar). Statistical significance: * p<0.05, ** p<0.001, *** p<0.0001.

We next performed cotransfection assays with POU4 expression vectors to test whether POU4 factors can modulate the *RIT2* promoter. We used SK-N-AS cells as host for these studies because i) the cells express endogenous *RIT2* at a detectable level (Figure 1B), indicating that they contain the transcriptional machinery for activating the *RIT2* promoter; ii) their endogenous *RIT2* expression is low (Figure 1B), suggesting that background activation of the *RIT2* promoter would be low; and iii) they express POU4 factors at low or undetectable levels as analyzed by RT-PCR (data not shown), suggesting that these cells might be responsive to increased expression of POU4 factors. The results of the cotransfection studies were somewhat complicated, with different POU4 factors and isoforms having different effects (Figure 6). The effects of POU4 factors were generally stronger with *RIT2*-1290/luciferase, as compared to *RIT2*-374/luciferase. With the *RIT2*-1290/luciferase construct, POU4F1-s, POU4F2-l, and POU4F3 all significantly increased reporter activity to different degrees, whereas POU4F2-s significantly repressed it, with POU4F1-l showing no effect. With the *RIT2*-374/luciferase construct, POU4F1-s still significantly increased reporter activity; however, the effects of POU4F2-l and POU4F3 were no longer significant. Both POU4F1-l and POU4F2-s significantly decreased reporter activity. In addition to POU4 factors alone, we also tested whether ISL1, a LIM homeodomain transcription factor expressed in RGCs [55,56], has an effect on the *RIT2* promoter modulation by POU4 factors. ISL1 by itself had no effect on either construct; however, ISL1 modestly but significantly enhanced the activity of POU4F2-l with both the −1290 and −374 constructs (Figure 6). The effect of ISL1 was also significant on the activity of POU4F1-s with the −374 construct, POU4F1-l with the −1290 construct, and POU4F2-s with both constructs. These results suggest that the POU4 factors can indeed modulate the *RIT2* promoter, and that various POU4 factors and their isoforms have distinct activities, which can be further modulated by the activity of ISL1.

## Discussion

In this paper, we have begun to explore the mechanisms mediating the neuron-specific expression of RIT2. We previously found by in situ hybridization that *Rit2* is expressed in RGCs [5], and here we confirmed the expression of *Rit2* in RGCs at the mRNA and protein levels. In addition, we showed for the first time RIT2 protein expression in non-RGC cells in the GCL and all three neuronal cells in the INL, i.e., horizontal, bipolar, and amacrine cells, suggesting that RIT2 is expressed in a broader set of neurons than previously identified. As for *Rit2* expression in photoreceptors, we obtained conflicting results with the two methods, RT-qPCR of LCM samples and immunohistochemistry of retinal sections, leaving the issue unresolved in the present study and requiring future investigation. Transient transfection analyses indicated that the human *RIT2* −1290 to +76 bp region could drive expression preferentially in differentiated neuronal cells, and that the −1290 to −374 bp region might contain elements involved in such expression. The next question was what transcription factors might mediate the neuronal activity of the *RIT2* upstream region. Because of our interest in RGCs, we were most interested in transcription factors expressed in these cells. Several observations suggested POU4 family members were interesting candidates. i) POU4 family members are expressed in RGCs, in which RIT2 is also highly expressed. ii) Our bioinformatic analysis indicated the presence of possible POU4 protein binding sites in the *RIT2* promoter. iii) Although the expression of RIT2 is broader than that of the POU4 family, RIT2 seems to be expressed in most neurons that express a POU4 factor [19,21,26-29]. iv) Similar to RIT2, POU4 factors have been implicated in neurite outgrowth. For example, induction of neurite outgrowth in ND7 neuronal cells by serum removal increases *Pou4f1* expression [57], and overexpression of POU4F1 in ND7 induces neurite outgrowth [58]. v) Furthermore, RIT2 was identified by a yeast two-hybrid screen as a protein that interacts with the N-terminus of POU4F1 [18]. These findings raised the interesting possibility that RIT2 might modulate POU4 factor activity and thus form a feedback or feed forward loop for autoregulation.

To test the hypothesis that POU4 factors may modulate *RIT2* expression, we examined all known POU4 factors and their isoforms, short and long forms of POU4F1 and POU4F2 [34,54]. These isoforms are derived from the same gene by alternative promoter usage and splicing. POU4F1-s and POU4F2-s are produced from a downstream intronic promoter, and therefore lack the N-terminus of their corresponding long forms. POU4F1-s and POU4F2-s are missing 84 and 97 N-terminal amino acids, respectively, and POU4F2-s contains nine unique N-terminal residues [34,54]. The relative proportion of the long and short forms of POU4F1 and POU4F2 varies in different parts of the nervous system [54,59]. Interestingly, differentiation-inducing stimuli such as cyclic adenosine monophosphate induce POU4F1-s mRNA in ND7 and primary dorsal root ganglion cells, paralleling the rise in total POU4F1, but barely increase or decrease POU4F1-l mRNA, resulting in the up to 10-fold increase in the ratio of the short to long form. For POU4F2, changes are in the opposite direction, i.e., differentiation stimuli decrease POU4F2-s mRNA, and the ratio of the short to long form decreases [54].

Of the six predicted POU4 protein binding sites, three showed detectable binding in EMSA, with the order of binding Site 2 > Site 6=Site 1. This order did not correlate well with the scores derived from the binding matrices used [27,50]; for example, scores for Site 2 ranked fourth with both matrices. Nevertheless, in cold oligomer competition EMSA the Matrix 1 consensus site (gcATAATTAAT) showed the strongest competition, which was tenfold stronger than Site 2, consistent with the finding that the Matrix 1 consensus site had the highest affinity among the elements tested [50]. However, in more complex in vivo situations, which involve chromatin structures and interaction with other DNA-binding proteins and cofactors, relatively low affinity sites can still be functionally significant through the mechanisms such as protein–protein interaction and cooperative binding. Also of interest, the different POU4 factors, and their isoforms, demonstrated distinct binding profiles. For example, while Probe 2 was bound well by all proteins, Probe 1 was bound only weakly, if at all, by POU4F2-l, and Probe 6 was weakly bound by POU4F2-l and POU4F3. Even proteins containing identical POU domains (e.g., POU4F2-s and POU4F2-l) showed differential binding, suggesting that protein regions outside the POU domain can modulate binding activity.

Further suggesting the complexity of DNA interactions with the POU4 family of proteins, our cotransfection studies showed that POU4 proteins not only can modulate the *RIT2* promoter but also have distinct activities. The data showed that, at least in some circumstances, POU4F1 and POU4F2 can have opposite effects, as can the respective isoforms. These results are consistent with several earlier reports describing differential activities of the POU4 factors. POU4F1 and POU4F3 can activate the α-internexin promoter, while POU4F2 represses it and can prevent activation by POU4F1 [60]. Overexpression of POU4F1 in ND7 cells induces neurite outgrowth and the expression of synaptic proteins [58]; in contrast, overexpression of POU4F2 reduces neurite outgrowth and synaptic vesicle-related gene expression by a stimulus that would normally induce differentiation [61]. Of interest, a single amino acid at position 22 in the POU homeodomain, valine in POU4F1 and isoleucine in POU4F2, determines the function of these factors. The mutation I22V converts POU4F2 from a repressor to an activator of the SNAP25 promoter; the converse mutation V22I alters POU4F1 from an activator to a repressor [62]. These results are consistent with our data in that the “pro-neuronal differentiation” forms of POU4F1 and POU4F2 (POU4F1-s and POU4F2-l) are the ones that positively modulate the *RIT2* promoter, suggesting that the neurite-promoting activity of these isoforms may, at least in part, be mediated by the upregulation of RIT2 expression.

The N-terminal domain of POU4F1 and POU4F2 likely mediates the differential activity of the isoforms, since this is the major difference between the molecules [63]. In addition to the conserved C-terminal POU domain that functions for DNA-binding and transcriptional activation, an N-terminal activation domain has been identified in all three POU4 factors [60,64,65]. Although the POU domain activates some targets, activation of a subset of target promoters requires the N-terminal domain [60,65-67]. In addition, as already described, RIT2 interacts with the N-terminal domain of POU4F1 in a yeast two-hybrid screen and several in vitro binding assays [18]. These published results together with our data suggest the possibility of a complex network of interactions between RIT2 and POU4 factors.

It is reported that POU4F2 and ISL1 synergistically regulate the expression of a common set of RGC-specific genes [55,56]. The cooperative function of POU4F2 and ISL1 seems critical for RGC development, as *Pou4f2* and *Isl1* double knockout mice showed a near complete loss of RGCs, a phenotype more severe than that of each single gene knockout [56]. In the present study, we found that ISL1 modestly but significantly enhanced *RIT2* promoter activation by POU4F2-l. In addition, we found significant effects of ISL1 on *RIT2* promoter modulation by other POU4 factors, depending on the combination of POU4 factors and the promoter constructs.

Although we feel the evidence presented makes a reasonable argument suggesting the existence of modulatory interactions between RIT2 and POU4 factors, some data, at least at first glance, do not suggest such interactions. To identify downstream targets of POU4 factors and ISL1, cDNA microarray analyses using mice with targeted disruption of *Pou4f1*, *Pou4f2*, or *Isl1* were performed [55,68,69]. These studies did not report changes in *Rit2* expression associated with *Pou4f1*, *Pou4f2*, or *Isl1* deletion. However, this finding does not rule out possible effects of these factors on *Rit2* because the studies cited analyzed only embryonic time points, and *Rit2* is expressed at much higher levels in differentiated tissues, in the brain as reported [5] and in the retina as our immunohistochemistry and western blot analysis revealed in this study. For genes that are expressed at low levels, microarray analysis is generally more variable, and as a result, it is more difficult to demonstrate small changes in expression. In addition, failure to observe an effect following knockout of a transcription factor can also be due to redundancy and possible compensation by other POU4 factors. Furthermore, the regulatory mechanism of *Rit2* expression might be different between embryonic and adult tissues due to possible changes in regulatory components, such as the availability of other factors, chromatin structure, and epigenetic state. Future studies, including examination of conditional *Pouf4* knockouts at postnatal stages, are needed to resolve these issues.

## Appendix 1. Primer sequences.

To access the data, click or select the words “Appendix 1.”

## Appendix 2. Gene Structure of RIT1 and RIT2.

To access the data, click or select the words “Appendix 2.”

## Appendix 3. RIT2 expression in the mouse retina at embryonic, newborn, and adult stages.

To access the data, click or select the words “Appendix 3.”

## Appendix 4. Bioinformatic prediction of POU4 protein binding sites in the RIT2 promoter.

To access the data, click or select the words “Appendix 4.”
